# FEV_1_ Is a Better Predictor of Mortality than FVC: The PLATINO Cohort Study

**DOI:** 10.1371/journal.pone.0109732

**Published:** 2014-10-06

**Authors:** Ana Maria B. Menezes, Rogelio Pérez-Padilla, Fernando César Wehrmeister, Maria Victorina Lopez-Varela, Adriana Muiño, Gonzalo Valdivia, Carmen Lisboa, José Roberto B. Jardim, Maria Montes de Oca, Carlos Talamo, Renata Bielemann, Mariana Gazzotti, Ruy Laurenti, Bartolomé Celli, Cesar G. Victora, for the PLATINO team

**Affiliations:** 1 Federal University of Pelotas, Post-graduate Program in Epidemiology, Pelotas, Brazil; 2 National Institute of Respiratory Diseases, Sleep Clinic and Pulmonary Physiology, Mexico City, Mexico; 3 Universidad de la Republica, Facultad de Medicina, Montevideo, Uruguay; 4 Centro Hospitalario Pereira Rossell, Montevideo, Uruguay; 5 Pontificia Universidad Católica de Chile, Santiago de Chile, Chile; 6 Federal University of São Paulo, São Paulo, Brazil; 7 Universidad Central de Venezuela, Facultad de Medicina, Caracas, Venezuela; 8 University of Sao Paulo, Sao Paulo, Brazil; 9 St. Elizabeth's Medical Center, Pulmonary and Critical Care, Boston, Massachusetts, United States of America; Clinica Universidad de Navarra, Spain

## Abstract

**Objective:**

To determine whether the presence of chronic obstructive lung disease (COPD) and reduction of lung function parameters were predictors of mortality in a cohort.

**Materials/Patients and Methods:**

Population based cohorts were followed in Montevideo, Santiago and Sao Paulo during 5, 6 and 9 years, respectively. Outcomes included all-cause, cardiovascular, respiratory and cancer mortality; exposures were COPD, forced expiratory volume in one second (FEV_1_) and forced vital capacity (FVC). Cox regression was used for analyses. Sensitivity, specificity, positive and negative predictive values, receiver operator characteristics curves and Youden's index were calculated.

**Results:**

Main causes of death were cardiovascular, respiratory and cancer. Baseline COPD was associated with overall mortality (HR = 1.43 for FEV_1_/FVC<LLN; 2.01 for GOLD 2-4; 1.46 for GOLD 1-4; 1.50 for FEV_1_/FEV_6_ <LLN). For cardiovascular mortality, significant associations were found with GOLD 2-4 (HR = 2.68) and with GOLD 1-4 (HR = 1.78) for both genders together (not among women). Low FEV_1_ was risk for overall and respiratory mortality (both genders combined). FVC was not associated with overall mortality. For most COPD criteria sensitivity was low and specificity high. The area under the curve for FEV_1_ was greater than for FVC for overall and cardiovascular mortality.

**Answer to the Question:**

COPD and low FEV_1_ are important predictors for overall and cardiovascular mortality in Latin America.

## Introduction

Over the next 20 years, a dramatic rise in chronic obstructive lung disease-related (COPD) morbi-mortality is expected worldwide [1]. In 2005, results from the first population-based multi-center study in Latin America (LA) – the PLATINO study – highlighted the high prevalence of COPD in this region [2].

The literature shows that COPD and poor lung function are important predictors for all-cause, cardiovascular and respiratory mortality[3]–[7], but there is controversy regarding which lung function parameter is the best predictor.

Most of the evidence comes from high-income countries [3]–[8]. Results from one of the few studies outside this more affluent population showed that forced expiratory volume in one second (FEV_1_) was an important predictor of overall and cancer mortality, but forced vital capacity (FVC) was not measured [9].

A recent publication presenting spirometric data from 17 countries concluded that lung function differs substantially between regions worldwide, and that such differences are not explained by variation in the distribution of height, age, gender, weight, residence, or educational level [10]. Compared with North America or Europe, FEV_1_ and FVC adjusted for height and gender were lower in several regions including South America [10]. In this study, there was no attempt to correlate lung function with mortality.

The present analyses are aimed at evaluating how reliable are COPD (according to different spirometric criteria) and lung function as predictors of overall and cause-specific mortality in three urban sites in LA, by following up a population based cohorts which were part of the PLATINO study.

## Methods

The detailed methods of the PLATINO baseline [11] and follow-up [12] are available elsewhere. Between 2003 and 2005, population-based surveys were conducted in five LA metropolitan areas: Sao Paulo (Brazil), Mexico City (Mexico), Montevideo (Uruguay), Santiago (Chile) and Caracas (Venezuela). We interviewed 1000 subjects aged 40 years or older in Sao Paulo, 1063 in Mexico City, 943 in Montevideo, 1208 in Santiago and 1357 in Caracas [2].

Information collected at baseline by means of a standardized questionnaire is available at: http://www.platino-alat.org.

Spirometry was undertaken in 99.0% of the sample using a portable, battery-operated, Easy One spirometer (ndd Medical Technologies, Switzerland); tests were performed at baseline and 15 minutes after 200 µg of salbutamol according to the American Thoracic Society (ATS) criteria of acceptability and reproducibility [13]. The quality control showed that over 90% of the tests fulfilled ATS criteria of quality [11].

Follow-up studies were conducted in Montevideo, Santiago and Sao Paulo 5, 6 and 9 years after the baseline surveys, respectively. Only individuals with valid spirometric data at baseline were eligible for follow-up.

The outcomes were all-cause mortality and mortality due to cardiovascular diseases, respiratory diseases and cancer. Lung cancer was considered among cancer deaths and not among the respiratory causes of mortality. Deaths were identified through the household interviews; for subjects known to have died, death certificates were obtained from the National Death Registry from each country. Quality of death certificates was evaluated by an expert (R.L.) who identified also the underlying cause of death. The percentage of ill-defined causes in the death certificates from Montevideo was higher than in the other sites (49.3% against 14.7% in Santiago and 11.1% in São Paulo). We opted to present the analyses on overall rates and those on causes of deaths for the three sites, and, as supplementary material the data without Montevideo (Table S9 in File S4).

Data analyses contained the description of the sample and the mortality incidence rates for site and by gender, and the crude and age-adjusted mortality rates using the WHO reference population [14]. The Kaplan-Meier method was used to derive survival curves.

Adjustment for age and country and for additional confounders was carried out using Cox regression, and hazard ratios (HR) were calculated for the following exposures: presence of COPD and lung function parameters. COPD was defined using several criteria: a) FEV_1_/FVC <lower limit of normal (LLN) [15] – the lower 5^th^ percentile for predicted post-bronchodilator (BD) FEV_1_/FVC based on PLATINO equations [16]; b) a ratio of the post-BD FEV_1_ over FVC below 0.70 (GOLD 1-4); c) the previous criteria plus a FEV_1_ below 80% predicted (GOLD 2-4) [1]; and d) FEV_1/_FVC_6_ <LLN – the lower 5^th^ percentile for predicted post-BD FEV_1_/FVC_6_ based on PLATINO equations [16]. The lung function parameters analyzed were: post-BD FEV_1_ (Liters) and FVC (Liters).

The main confounders were: gender, age (40–49, 50–59 and ≥ 60 years of age) and age as a quadratic term, schooling (0–4 and ≥ 5 years), current status of smoking (never, former and current) and pack-years (average number of cigarettes packs/day times the number of years smoked). A comorbidity score was calculated by counting the number of self-reported doctor diagnosed diseases (we added one point for each comorbidity): heart disease, hypertension, stroke, diabetes, lung cancer, ulcer and tuberculosis. Health status was assessed using the physical domain from the Short-Form-12 questionnaire (continuous variable) [17]. Weight and height were measured and body mass index (BMI) was calculated as the ratio weight (kg)/height (m)^2^ (kg/m^2^) (categorized into two groups: normal/underweight and overweight/obesity). All the analyses took into account the sampling scheme with adjustment for “country” and were stratified by gender.

Sensitivity, specificity, positive (PPV) and negative predictive values (NPV) were calculated for overall and all-causes mortality for all sites, according to the different criteria for COPD.

Additionally, receiver operating characteristics (ROC) curves stratified by gender were used to quantify whether FEV_1_ or FVC was the best predictor for all-cause mortality and specific causes mortality. The Youden's index (sensitivity + specificity – 1) was used as a measure of overall diagnostic effectiveness representing the maximum vertical distance between the ROC curve and the diagonal line [18]. This index was used to define the optimal cut-off point for both lung function parameters. To standardize mortality analyses across sites, we took the shortest duration of follow up (5.3 years in Montevideo) and used the same period in the other two sites.

Ethical approval was obtained by local Institutional Review Boards at baseline and at follow-up.

## Results

There were 885 eligible individuals for follow-up in Montevideo, 1173 in Santiago and 963 in Sao Paulo. Information was obtained for 85.6%, 84.7% and 77.7%, respectively. These included 71 deaths in Montevideo, 95 in Santiago and 135 in Sao Paulo. Death certificates were successfully obtained for 76.1% of the deaths in Montevideo, 88.3% in Santiago and 91.8% in Sao Paulo [12].

Table 1 describes the baseline characteristics of the sample from the three sites combined and the number of deaths within the follow-up period. Women were older and had less schooling than men; around 3/4 of men and half of the women were smokers. Deaths were more frequent among subjects who were older, had less schooling and among those with normal BMI more noticeable for men than for women. Among those who died, both genders, the mean comorbidity score was higher, while lung function parameters and health status were lower. Follow up rates for each category of independent variables were around 80% (Table 1). More detailed information is available elsewhere [12].

**Table 1:** Baseline characteristics for all sites (Montevideo, Santiago and Sao Paulo), number of deaths and follow up rates (The PLATINO Study).

	Males		Females		
	Eligible subjects	Deaths	Eligible subjects	Deaths	Follow up rates (all sample)
Variables	N (%)	N (rate/1,000)	N (%)	N (rate/1,000)	%
Age					
40–49	432 (34•8)	15 (34)	585 (32•9)	12 (20)	79•2
50–59	392 (31•6)	30 (75)	530 (29•8)	29 (54)	82•3
60+	418 (33•6)	101 (232)	664 (37•3)	114 (163)	83•5
					
Schooling					
5+	895 (72•1)	76 (83)	1221 (68•9)	63 (50)	81•9
0–4	347 (27•9)	70 (194)	551 (31•1)	92 (158)	81•1
					
Current smoker					
No	299 (24•1)	31 (100)	867 (48•7)	77 (85)	81•4
Yes	941 (75•9)	115 (119)	912 (51•3)	78 (84)	81•8
					
BMI					
Normal/Underweight	401 (32•3)	67 (161)	512 (28•9)	48 (90)	80•0
Overweight	839 (67•7)	79 (92)	1263 (71•1)	107 (82)	82•4
					
COPD					
GOLD 1-4	281 (22•6)	62 (221)	243 (13•7)	44 (181)	83•2
FEV_1_/FVC <LLN	217 (17•8)	44 (203)	164 (9•5)	19 (116)	81•6
GOLD 2-4	102 (8•2)	33 (324)	99 (5•6)	19 (192)	85•1
FEV_1_/FVC_6_ <LLN	136 (11•2)	32 (235)	115 (6•7)	16 (139)	82•5
					
Pack years [mean (SD)]	17•62 (22•83)	28•5 (33•1)	7•29 (13•51)	8•62 (18•78)	12•2 (19•8)
					
Comorbidities score [mean (SD)]	0•79 (0•86)	1•25 (1•15)	1•09 (0•98)	1•46 (1•15)	1•03 (0•98)
					
SF-12 physical domain [mean (SD)]	52•66 (6•85)	49•18 (9•67)	49•21 (9•39)	44•86 (11•16)	50•0 (8•98)
					
Lung function (post-BD)					
FEV_1_ (liters) [mean (SD)]	3•28 (0•71)	2•63 (0•85)	2•33 (0•55)	1•80 (5•6)	2•66 (0•80)
FVC (liters) [mean (SD)]	4•30 (0•82)	3•81 (0•89)	2•96 (0•62)	2•41 (6•7)	3•47 (0•98)

Note: data for continuous variables (pack-year smoking, comorbidities score, SF-12 and lung function parameters) are presented as mean (standard deviation (SD) in survival individuals (eligible subjects column) and mean (SD) in those who died (deaths column).

FEV_1_ – forced expiratory volume in one second; FVC – forced vital capacity; FVC_6_ - forced vital capacity in 6 seconds; BD – bronchodilator.

**Figure 1:**
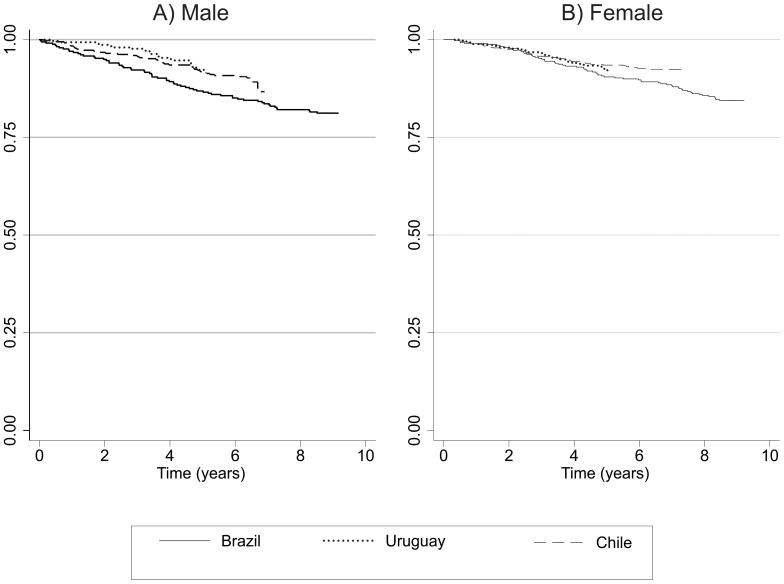
Survival curves according to the Kaplan-Meier method by site and gender. The PLATINO Study.

Figure 1 presents crude survival curves using the Kaplan Meier method. Among men, survival rates appeared to be lower in Sao Paulo as compared to the other cities from year 2 onwards, but this difference was not statistically significant (log rank test p = 0.11). Among women, the survival curves were similar in all sites (log rank test p = 0.21).

Table 2 displays the crude and age-standardized mortality rates (SMR) by country and gender. In agreement with the survival curves, SMR were similar in Montevideo and Santiago, but appeared to be slightly higher in Sao Paulo. Most SMR tended to be lower in women than in men. Cardiovascular deaths were the leading causes of death for both genders in Santiago and Sao Paulo, and for men in Montevideo. Cancer and respiratory deaths were the next two most common causes of death overall.

**Table 2:** Crude and age-standardized (WHO population) incidence density mortality rate (by 1,000 persons-year), by site and gender (The PLATINO Study).

	Number of deaths		Crude Rate			Standardized rate (WHO population)		
	Males	Females	Males	Females	Ratio M/F	Males	Females	Ratio M/F
***Montevideo (Uruguay)***								
Overall	35	36	24•3	17•4	1•4	22•4	13•9	1•6
Cardiovascular	6	5	4•2	2•4	1•8	4•0	1•6	2•5
Cancer	4	9	2•8	4•3	0•7	2•5	3•7	0•7
Respiratory	2	1	1•4	0•5	2•8	1•2	0•4	3•0
Metabolic and endocrine	0	0	0	0	0	0	0	0
Others	2	4	1•3	1•9	0•7	1•1	1•9	0•6
Ill defined	21	17	14•6	8•2	1•8	13•6	6•3	2•2
***Santiago (Chile)***								
Overall	45	50	19•8	13•1	1•5	22•3	13•5	1•7
Cardiovascular	11	19	5•3	5•0	1•1	6•3	5•2	1•2
Cancer	9	12	3•9	3•1	1•3	4•4	3•4	1•3
Respiratory	6	4	2•4	1•0	2•5	3•0	1•1	2•7
Metabolic and endocrine	2	2	0•9	0•5	1•7	1•1	0•5	2•2
Others	9	6	3•9	1•6	2•5	4•4	1•5	2•9
Ill defined	7	7	3•1	1•8	1•7	3•1	1•8	1•7
***Sao Paulo (Brazil)***								
Overall	66	69	25•1	18•4	1•4	31•9	20•5	1•6
Cardiovascular	25	22	9•5	6•8	1•4	11•9	7•7	1•6
Cancer	9	16	3•4	4•6	0•7	3•8	4•9	0•8
Respiratory	7	8	2•7	2•1	1•2	4•9	2•5	2•0
Metabolic and endocrine	5	7	1•9	2•1	0•9	2•6	2•4	1•1
Others	15	6	5•7	1•8	3•1	6•9	2•0	3•4
Ill defined	5	10	1•9	3•1	0•6	1•8	3•4	0•5

Table 3 presents the age and site-adjusted hazard ratio (HR) for mortality and after additional adjustment for other confounders according to the presence of COPD (defined by different spirometric criteria) and lung function in the baseline. With the exception of cancer deaths, all HR for men were greater than one, but results for women were less consistent. HRs adjusted for confounders for which the 95% CI did not include unity are described below. For the whole sample, being diagnosed with COPD as defined by the FEV_1_/FVC<LLN criterion at baseline was associated with a 43% increase in overall mortality; the corresponding increases were 101% for GOLD 2-4, 46% for GOLD 1-4 and 50% according to FEV_1_/FEV_6_ <LLN. For cardiovascular mortality, significant associations were found with the GOLD 2-4 (168% increase) and with GOLD 1-4 (78%). All these associations were significant for men but not among women.

**Table 3:** Hazard ratio (HR) according to different criteria of Chronic Obstructive Pulmonary Disease (COPD) and mortality for all sites (The PLATINO Study).

	All causes		Cardiovascular		Respiratory		Cancer	
	Adjusted*	Adjusted**	Adjusted*	Adjusted**	Adjusted*	Adjusted**	Adjusted*	Adjusted**
	HR (95% CI)	HR (95% CI)	HR (95% CI)	HR (95% CI)	HR (95% CI)	HR (95% CI)	HR (95% CI)	HR (95% CI)
***Both Genders***								
LLN	1•82 (1•35; 2•44)	1•43 (1•05; 1•94)	1•88 (1•03; 3•44)	1•50 (0•79; 2•83)	4•78 (1•35; 16•93)	3•43 (0•85; 13•88)	0•80 (0•31; 2•10)	0•68 (0•25; 1•83)
GOLD 2-4	2•54 (1•76; 3•67)	2•01 (1•39; 2•88)	3•09 (1•59; 6•02)	2•68 (1•35; 5•33)	3•13 (1•05; 9•32)	1•49 (0•51; 4•37)	1•19 (0•42; 3•39)	1•06 (0•38; 3•01)
GOLD 1-4	1•77 (1•39; 2•24)	1•46 (1•13; 1•88)	2•19 (1•32; 3•62)	1•78 (1•05; 3•04)	1•82 (0•78; 4•27)	1•09 (0•43; 2•73)	1•57 (0•80; 3•06)	1•41 (0•71; 2•82)
FEV_1_/FEV_6_<LLN	1•90 (1•33; 2•70)	1•50 (1•05; 2•15)	2•28 (1•14; 4•54)	1•86 (0•95; 3•62)	4•48 (1•18; 16•96)	3•35 (0•77; 14•57)	0•47 (0•11; 2•04)	0•41 (0•10; 1•68)
***Males***								
LLN	2•06 (1•38; 3•08)	1•70 (1•13; 2•57)	2•43 (1•17; 5•03)	2•10 (0•98; 4•52)	4•57 (1•26; 16•53)	2•44 (0•54; 11•00)	0•55 (0•12; 2•53)	0•51 (0•11; 2•40)
GOLD 2-4	3•01 (1•86; 4•88)	2•50 (1•58; 3•95)	3•37 (1•37; 8•27)	4•05 (1•54; 10•65)	6•06 (1•52; 24•21)	3•77 (1•02; 13•87)	1•10 (0•25; 4•76)	1•04 (0•23; 4•73)
GOLD 1-4	1•85 (1•29; 2•65)	1•52 (1•06; 2•20)	2•13 (0•96; 4•77)	1•76 (0•73; 4•24)	3•30 (0•96; 11•35)	1•70 (0•33; 8•72)	1•25 (0•43; 3•63)	1•23 (0•44; 3•47)
FEV_1_/FEV_6_<LLN	2•17 (1•38; 3•44)	1•89 (1•20; 2•95)	3•42 (1•54; 7•59)	3•22 (1•45; 7•16)	4•42 (1•11; 17•66)	2•71 (0•68; 10•76)	0•44 (0•06; 3•58)	0•42 (0•06; 3•02)
***Females***								
LLN	1•30 (0•80; 2•11)	0•97 (0•57; 1•64)	1•10 (0•39; 3•13)	0•78 (0•24; 2•48)	1•44 (0•24; 8•74)	4•89 (0•80; 29•78)	1•09 (0•32; 3•75)	0•99 (0•28; 3•52)
GOLD 2-4	1•84 (1•04; 3•24)	1•35 (0•74; 2•47)	2•66 (0•98; 7•20)	2•06 (0•78; 5•46)	--	--	1•23 (0•28; 5•38)	1•13 (0•29; 4•43)
GOLD 1-4	1•51 (1•07; 2•14)	1•27 (0•86; 1•87)	2•09 (1•04; 4•20)	1•73 (0•84; 3•56)	0•39 (0•13; 1•21)	0•23 (0•05; 1•14)	1•85 (0•74; 4•59)	1•72 (0•69; 4•33)
FEV_1_/FEV_6_<LLN	1•37 (0•79; 2•37)	0•98 (0•53; 1•79)	1•02 (0•29; 3•58)	0•64 (0•17; 2•44)	1•51 (0•25; 9•08)	5•06 (1•10; 23•30)	0•51 (0•06; 4•07)	0•45 (0•06; 3•19)

* Adjusted for age and country.

** Adjusted for age, country and confounders (schooling, smoking status, pack-years smoking, quality of life, BMI and comorbidities score).

Patterns for cardiovascular deaths were similar to those of overall mortality, although some of the associations for both genders and for men were no longer significant, possibly due to small numbers. None of the associations for women were significant. Due to the small number of respiratory deaths, patterns are not as clear, but most HR were increased. There were no significant associations between COPD under any definition with cancer deaths. We performed a supplementary analysis for the outcomes “respiratory mortality including lung cancer” and “for lung cancer as a single outcome category” according to different spirometric criteria of COPD only for lung cancer mortality (Table S1 in File S1). Most HR are increased in the analysis of the outcome “mortality for lung cancer”, although the results did not reach a statistical significance.

Table 4 shows the predictive ability of the COPD diagnostic criteria, as currently defined (for all-cause mortality). Results are shown for all sites combined. The GOLD 1-4 criteria show the highest sensitivity (40%) for mortality whereas the other three criteria ranged from 20% to 27%. In terms of specificity, the highest was achieved by GOLD 2-4 (95%) followed by FEV_1_/FEV_6_ (92%) whereas the other criteria had values for specificity that were below 90%. PPV were below 25% for all criteria, and NPV were all above 90%. Tables S2, S3 and S4 in File S2 show the cause of death and gender stratified results, which are well in line with the all-cause results described above.

**Table 4:** Validity parameters for prediction of overall mortality for all sites according to different criteria of Chronic Obstructive Pulmonary Disease (COPD) (The PLATINO Study).

Diagnostic classification	Sensitivity % (95%CI)	Specificity % (95%CI)	Positive predictive value % (95%CI)	Negative predictive value % (95%CI)	Youden's index
***Both Genders***					
LLN	26•7 (21•0; 33•0)	88•2 (86•9; 89•4)	15•5 (12•0; 19•5)	93•7 (92•7; 94•6)	14•9
GOLD 2-4	20•7 (15•7; 26•4)	94•5 (93•6; 95•4)	24•4 (18•6; 30•9)	93•3 (92•3; 94•2)	15•2
GOLD 1-4	40•1 (33•8; 46•6)	84•6 (83•2; 85•9)	18•1 (14•9; 21•7)	94•3 (93•3; 95•2)	24•7
FEV_1_/FEV_6_	20•4 (15•3; 26•3)	92•4 (91•4; 93•4)	17•9 (13•4; 23•2)	93•5 (92•5; 94•4)	12•8
***Males***					
LLN	36•9 (28•0; 46•6)	84•1 (81•8; 86•2)	18•9 (13•9; 24•7)	93•0 (91•2; 94•5)	21•0
GOLD 2-4	26•1 (18•4; 34•9)	93•7 (92•1; 95•0)	30•4 (21•7; 40•3)	92•3 (90•6; 93•8)	19•8
GOLD 1-4	47•1 (37•8; 56•4)	80•0 (77•5; 82•3)	19•9 (15•4; 25•1)	93•4 (91•7; 94•9)	27•1
FEV_1_/FEV_6_	27•0 (19•0; 36•3)	90•4 (88•5; 92•1)	22•1 (15•4; 30•0)	92•5 (90•8; 94•0)	17•4
***Females***					
LLN	16•4 (10•0; 24•6)	91•0 (89•4; 92•3)	11•0 (6•6; 16•8)	94•1 (92•8; 95•2)	7•4
GOLD 2-4	15•3 (9•3; 23•0)	95•1 (94•0; 96•1)	18•2 (11•1; 27•2)	94•0 (92•8; 95•1)	10•4
GOLD 1-4	33•1 (24•7; 42•3)	87•7 (86•0; 89•3)	16•0 (11•7; 21•3)	94•9 (93•6; 95•9)	20•8
FEV_1_/FEV_6_	13•6 (7•8; 21•5)	93•8 (92•5; 94•9)	13•0 (7•5; 20•6)	94•1 (92•8; 95•2)	7•4

Youden's index: sensitivity + specificity – 1.

Out of the spirometric measures in the adjusted analysis, an increase of 1 Liter in the FEV_1_ showed a HR of 0.62 (95%CI 0.46; 0.83) for overall mortality, 0.38 (95%CI 0.23; 0.61) for cardiovascular mortality and 0.26 (95%CI 0.08; 0.80) for respiratory mortality in both genders combined. FVC did not show a significant association with overall mortality (HR = 0.81, 95%CI 0.61; 1.07) for both genders combined, although it was associated with lower cardiovascular mortality (HR = 0.51, 95%CI 0.33; 0.78) (data not shown).

**Figure 2:**
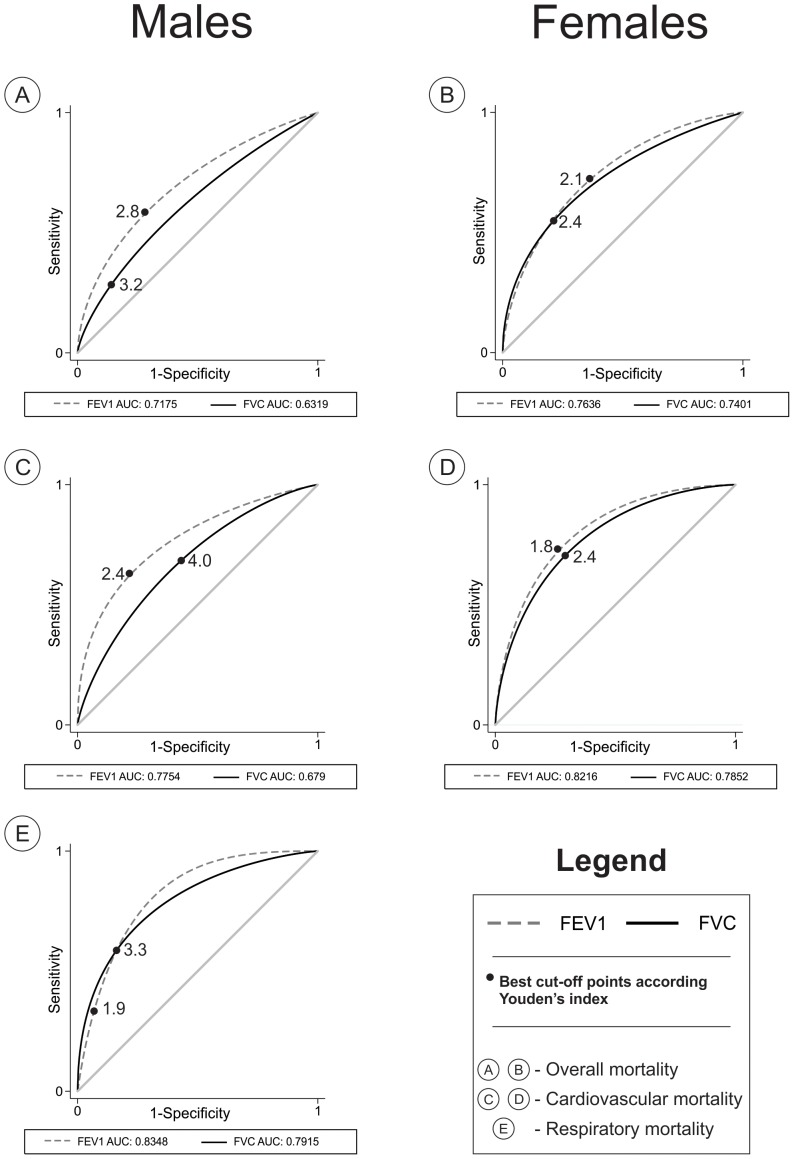
Receiver operator characteristic (ROC) curves for mortality according to the forced expiratory volume in the first second (FEV_1_) and forced vital capacity (FVC), stratified by gender. The PLATINO Study.

Figure 2 shows the ROC curves for FEV_1_ and FVC stratified by gender according to all-cause, cardiovascular and respiratory mortality. Due to the small number of respiratory deaths within the 5.3 years period among women, ROC curves were not calculated. In all other comparisons, the area under the curve for FEV_1_ was greater than for FVC, even though some of the differences were not statistically significant. The p values for all-cause mortality in men and women were <0.001 and 0.04, respectively, and for cardiovascular mortality these were 0.002 and 0.07, respectively. The p value for comparing the two curves for respiratory mortality in men was 0.49.

We used the Youden's index to estimate the cut off points associated with the optimal combination of specificity and sensitivity for the two spirometric measures. These are displayed as dots in Figure 2 (full results available in Tables S5, S6, S7 and S8 in File S3). The cutoffs varied according to the outcomes; for FEV_1_ the cut-off point ranged from 1.9 L for respiratory mortality to 2.8 L for overall mortality, while the FVC cut-off ranged from 3.2 for all causes mortality to 4.0 for cardiovascular mortality. Similar variations were observed among women.

## Discussion

These prospective analyses describe mortality rates according to COPD status and lung function measured at baseline in population-based samples of adults living in three LA metropolises, with follow up periods ranging from five to nine years. Standardized methods were used to classify subjects at baseline, and follow up rates were above 75%.

Our findings on mortality rates were consistent with the literature. Deaths were more common among men than women, and persons from Sao Paulo had higher rates than those from Santiago and Montevideo. According to the Global Burden of Disease Study 2010 [19], life expectancy in Brazilian males is 70.5 years compared to 77.7 years for females, and the corresponding figures for Uruguay are 72.6 y and 80.4 y, and for Chile 75.5 y vs. 81.5 y [19]. Cardiovascular, respiratory and cancer were the main causes of death, in accordance with the literature from LA [19], [20].

Poor lung function is an important predictor of overall, cardiovascular and respiratory mortality [3], [5]–[8], but there is controversy regarding which parameter shows the greatest predictive ability [3], [4], [21]–[25]. Most of the evidence is derived from cohorts studies in high-income settings, such as the Atherosclerosis Risk in Communities (ARIC), the Copenhagen City Heart, the Cardiovascular Health and the NHANES (National Health and Nutrition Examination Survey) studies [3], [6], [23], [24], [26]. In addition, none of these studies used bronchodilators prior to spirometric evaluation, and this may lead to misclassification of COPD status [27]. An earlier publication of the PLATINO baseline survey showed that the prevalence of airflow obstruction measured by the GOLD 1-4 criteria was 35% higher before bronchodilator use, compared to prevalence after its use [15].

In the analyses adjusted by site and age, the four spirometric tested diagnostic criteria for COPD predicted all-cause, cardiovascular and respiratory mortality for both genders combined and for men. For women, results were less consistent in terms of statistical significance, but COPD tended to be associated with increased risk. After adjustment for several confounders, the HR tended to be attenuated, but in general COPD was associated with greater risk of overall and cardiovascular mortality. Including lung cancer among the respiratory causes of mortality the results were quite similar.

The lack of significance in the associations with respiratory causes must be interpreted in light of the small number of deaths in this category and as a result of the low statistical power. There were no associations between COPD status and cancer mortality; however, in the supplementary analysis with “lung cancer mortality” as a single outcome according to the different spirometric criteria of COPD, we were able to detect an increase in all HR although the results did not reach statistical significance due to the small sample size (only 10 deaths by lung cancer out of 46 cancer deaths).

These findings from the survival analyses are reflected by the calculations of specificity and sensitivity. The latter tended to be very low for all four diagnostic criteria, being a little better for GOLD 1-4 (40% for overall mortality). Specificity on the other hand tended to be high for the four criteria, particularly GOLD 2-4. Consistently, PPV were low, and NPV were high. None of the four diagnostic criteria of COPD was sufficiently sensitive to be recommended as a predictor of mortality rates.

Regarding the lung function parameters, FEV_1_ was clearly a better predictor of mortality than FVC; even after adjusting for potential confounders for mortality, low FEV_1_ remained as a significant predictor for overall mortality in both genders and for cardiovascular mortality in men. These results were confirmed by the ROC curves showing that the area under the curve for FEV_1_ was significantly greater than that for FVC, again for all-cause mortality in both genders and for cardiovascular mortality in men. The lack of significant associations with respiratory mortality is likely due to the small number of deaths, particularly among women. We attempted to suggest cut-off points for FEV_1_ or FVC for predicting mortality, but this was not possible given that the ROC curves vary by gender and mortality cause.

The link between poor lung function and COPD as predictors of all-cause and cardiovascular mortality is supported by studies suggesting an interplay of systemic inflammation with airflow obstruction in the development of cardiac disease [3], [6]. Sin and Man [28] evaluating the potential role of systemic inflammation in COPD subjects showed the presence of systemic inflammation even in moderate COPD.

Some limitations of the present study should be mentioned. Quality of death certificates is very heterogeneous globally, particularly in low and middle-income countries [29]. In the three sites, deaths were ascertained directly by interviewing family members, but causes of death relied on death certificates. This was particularly problematic in Montevideo where death certificates were manually searched, which probably explains the lower percentage of those which were located (76.1%) compared to Santiago and Sao Paulo (88.3% and 91.8%, respectively). Another limitation was the poor quality of the information on causes of death in Montevideo with nearly 50% of certificates reporting ill-defined causes. Due to this limitation, we performed two sets of cause-specific analyses for hazard ratios, one including the three sites and another excluding Montevideo. Both sets of results were very similar (results shown as table S8 in File S3).

The fact that we have only two points in time for lung function parameters poses some statistical limitation for measuring the association of lung function decline and mortality; we hope to have a third follow-up visit in the PLATINO study and then we will be able of carrying out more robust analysis for lung function decline and its association with mortality.

Someone could also argue that categorizing subjects into racial/ethnical groups is not always a good proxy for genetic background. Some authors found a good correlation between self-defined race with inferred genetic clusters [30], [31], but the opposite has also been found [32] and the possible lung function misestimating using predictive equations based on self-reported race alone [33]. We did not perform the genomic ancestry analysis of our population as in most of the studies, but data not yet published from Brazil showed that self-report skin color has a good correlation with genomic ancestrality.

In spite of these limitations, this study has several strengths. It is the first prospective population-based study aimed at evaluating COPD (defined by different spirometric criteria) and pulmonary function as predictors of mortality in LA. The high follow up rates and the quality of the post-bronchodilator spirometry data reinforce the present findings. The consistency of the results between the several sets of analyses presented above is also reassuring.

COPD increases the risk of mortality in middle income countries consistent with previous studies mainly from developed countries. Our results show that FEV_1_ predicts mortality better than FVC in a population based cohort contributing to still an area of debate in the literature. Some of the findings in the literature come from specific groups of subjects and cannot be extrapolated to the general population; we believe that population based studies are the most reliable sources for drawing conclusions on the best lung function parameter to predict mortality. Burney found that survival was strongly associated with FVC adjusted for FEV_1_, but not the other way round in asymptomatic subjects from the ARIC study [26]; this group nevertheless had a substantial number of people with airway obstruction according to the author; interestingly, FEV_1_ was a better predictor for survival when analysis was carried out among the symptomatic group. The conclusion of the analysis cannot be applied to the general population because the studied sample was not representative of the general population. Another paper [34] showed that addition of FVC with the Framingham Risk Score offered significant incremental prediction of mortality in the intermediate risk group; the author also mentions that FEV_1_ relationships with mortality were weaker (data not shown in the paper); again, the analyzed sample was comprised of never-smokers subjects and without COPD diagnosis, therefore it is not a population based sample.

The importance of COPD and lung function as risk factors for mortality reinforces the need for strengthening preventive efforts, in particular tobacco control measures given the importance of smoking as identified in the original PLATINO findings [2] as well as in the ample global literature.

## Supporting Information

File S1 **Table S1.** Hazard ratio (HR) according to different criteria of Chronic Obstructive Pulmonary Disease (COPD) and mortality by respiratory diseases including lung cancer and by lung cancer only for all sites. The PLATINO Study.
(DOC)

File S2 **Table S2.** Validity parameters for prediction of cardiovascular mortality for all sites, according to different criteria of Chronic Obstructive Pulmonary Disease (COPD). The PLATINO Study. **Table S3.** Validity parameters for prediction of respiratory mortality for all sites, according to different criteria of Chronic Obstructive Pulmonary Disease (COPD). The PLATINO Study. **Table S4.** Validity parameters for prediction of cancer mortality for all sites, according to different criteria of Chronic Obstructive Pulmonary Disease (COPD). The PLATINO Study.
(DOC)

File S3 **Table S5.** Best cut-off points of forced expiratory volume in 1 second (FEV_1_) and forced vital capacity (FVC) to predict overall mortality in individuals belonging to the Platino Study. **Table S6.** Best cut-off points of forced expiratory volume in 1 second (FEV_1_) and forced vital capacity (FVC) to predict mortality by cardiovascular diseases in individuals belonging to the Platino Study. **Table S7.** Best cut-off points of forced expiratory volume in 1 second (FEV_1_) and forced vital capacity (FVC) to predict mortality by respiratory diseases in individuals belonging to the Platino Study. **Table S8.** Best cut-off points of forced expiratory volume in 1 second (FEV_1_) and forced vital capacity (FVC) to predict mortality by cancer in individuals belonging to the Platino Study.
(DOC)

File S4 **Table S9.** Hazard ratio between Chronic Obstructive Pulmonary Disease criteria and mortality in all sites from the PLATINO Study, except Montevideo.
(DOC)
