# Supplementary material for: FEV1 Is a Better Predictor of Mortality than FVC: The PLATINO Cohort Study
Source: PLoS One. 2014 Oct 6;9(10):e109732. doi: 10.1371/journal.pone.0109732 (PMC4186841; doi:10.1371/journal.pone.0109732)
Supplement: File S1 — Table S1. Hazard ratio (HR) according to different criteria of Chronic Obstructive Pulmonary Disease (COPD) and mortality by respiratory diseases including lung cancer and by lung cancer only for all sites. The PLATINO Study. (DOC) [file pone.0109732.s001.doc]

**Table S1.** Hazard ratio (HR) according to different criteria of Chronic Obstructive Pulmonary Disease (COPD) and mortality by respiratory diseases including lung cancer and by lung cancer only for all sites. The PLATINO Study.

|  | **Respiratory Diseases + Lung Cancer** | | **Lung Cancer** | |
| --- | --- | --- | --- | --- |
|  | **Adjusted*** | **Adjusted**** | **Adjusted*** | **Adjusted**** |
|  | **HR (95% CI)** | **HR (95% CI)** | **HR (95% CI)** | **HR (95% CI)** |
| ***Both Genders*** |  |  |  |  |
| LLN | 3.36 (1.56; 7.25) | 2.07 (0.95; 4.51) | 1.89 (0.36; 9.89) | 0.88 (0.16; 4.87) |
| GOLD 2-4 | 3.91 (1.99; 7.70) | 2.26 (1.16; 4.38) | 6.02 (1.39; 26.00) | 3.52 (0.75; 16.63) |
| GOLD 1-4 | 1.80 (0.96; 3.37) | 1.15 (0.62; 2.16) | 2.02 (0.46; 8.87) | 1.03 (0.24; 4.35) |
| FEV_1_/FEV_6_<LLN | 4.02 (1.78; 9.08) | 2.55 (1.09; 6.00) | 3.25 (0.59; 17.92) | 1.62 (0.28; 9.45) |
| ***Males*** |  |  |  |  |
| LLN | 3.14 (1.36; 7.23) | 2.10 (0.85; 5.19) | 1.26 (0.13; 12.59) | 1.46 (0.14; 14.93) |
| GOLD 2-4 | 5.41 (2.38; 12.26) | 3.84 (1.57; 9.42) | 4.70 (0.86; 25.61) | 4.70 (0.97; 22.80) |
| GOLD 1-4 | 2.37 (1.08; 5.21) | 1.52 (0.60; 3.85) | 1.35 (0.24; 7.43) | 1.60 (0.32; 7.92) |
| FEV_1_/FEV_6_<LLN | 4.04 (1.63; 10.0) | 2.53 (0.95; 6.74) | 2.35 (0.21; 25.65) | 2.83 (0.31; 25.68) |
| ***Females*** |  |  |  |  |
| LLN | 1.86 (0.44; 7.80) | 1.46 (0.25; 8.69) | 3.02 (0.28; 32.41) | 2.15 (0.28; 16.38) |
| GOLD 2-4 | 0.73 (0.08; 6.88) | 0.16 (0.00; 12.9) | 7.50 (0.56; 101.32) | 2.98 (0.23; 37.78) |
| GOLD 1-4 | 0.58 (0.17; 1.91) | 0.32 (0.07; 1.48) | 3.00 (0.21; 42.46) | 1.65 (0.11; 24.63) |
| FEV_1_/FEV_6_<LLN | 2.30 (0.49; 10.8) | 1.84 (0.32; 10.5) | 5.64 (0.47; 68.14) | 3.47 (0.37; 32.29) |

* Adjusted for age and country.

** Adjusted for age, country and confounders (schooling, smoking status, pack-years smoking, quality of life, BMI and comorbidities score).
