# Supplementary material for: FEV1 Is a Better Predictor of Mortality than FVC: The PLATINO Cohort Study
Source: PLoS One. 2014 Oct 6;9(10):e109732. doi: 10.1371/journal.pone.0109732 (PMC4186841; doi:10.1371/journal.pone.0109732)
Supplement: File S2 — Table S2. Validity parameters for prediction of cardiovascular mortality for all sites, according to different criteria of Chronic Obstructive Pulmonary Disease (COPD). The PLATINO Study. Table S3. Validity parameters for prediction of respiratory mortality for all sites, according to different criteria of Chronic Obstructive Pulmonary Disease (COPD). The PLATINO Study. Table S4. Validity parameters for prediction of cancer mortality for all sites, according to different criteria of Chronic Obstructive Pulmonary Disease (COPD). The PLATINO Study. (DOC) [file pone.0109732.s002.doc]

**Table S2.** Validity parameters for prediction of cardiovascular mortality for all sites, according to different criteria of Chronic Obstructive Pulmonary Disease (COPD). The PLATINO Study.

| **Diagnostic classification** | **Sensitivity**  **% (95%CI)** | **Specificity**  **% (95%CI)** | **Positive predictive value**  **% (95%CI)** | **Negative predictive value**  **% (95%CI)** | **Youden’s**  **index** |
| --- | --- | --- | --- | --- | --- |
| ***Both Genders*** |  |  |  |  |  |
| LLN | 35.5 (23.7; 48.7) | 87.5 (86.3; 88.7) | 5.8 (3.7; 8.6) | 98.4 (97.9; 98.9) | 23.0 |
| GOLD 2-4 | 31.3 (20.6; 43.8) | 93.9 (93.0; 94.7) | 10.4 (6.6; 15.5) | 98.4 (97.8; 98.8) | 25.2 |
| GOLD 1-4 | 52.2 (39.7; 64.6) | 83.4 (82.0; 84.7) | 6.7 (4.7; 9.2) | 98.7 (98.2; 99.1) | 35.6 |
| FEV_1_/FEV_6_ | 29.0 (18.2; 41.9) | 91.9 (90.8; 92.9) | 7.2 (4.3; 11.1) | 98.4 (97.8; 98.8) | 20.9 |
| ***Males*** |  |  |  |  |  |
| LLN | 50.0 (31.9; 68.1) | 83.0 (80.8; 85.1) | 7.4 (4.3; 11.7) | 98.4 (97.4; 99.1) | 33.0 |
| GOLD 2-4 | 35.3 (19.7; 53.5) | 92.5 (90.9; 94.0) | 11.8 (6.2; 19.6) | 98.1 (97.1; 98.8) | 27.8 |
| GOLD 1-4 | 58.8 (40.7; 75.4) | 78.3 (75.9; 80.6) | 7.1 (4.4; 10.8) | 98.5 (97.6; 99.2) | 37.1 |
| FEV_1_/FEV_6_ | 40.6 (23.7; 59.4) | 89.6 (87.7; 91.3) | 9.6 (5.2; 15.8) | 98.2 (97.3; 98.9) | 30.2 |
| ***Females*** |  |  |  |  |  |
| LLN | 20.0 (7.7; 38.6) | 90.7 (89.2; 92.0) | 3.7 (1.4; 7.8) | 98.5 (97.7; 99.0) | 10.7 |
| GOLD 2-4 | 27.3 (13.3; 45.5) | 94.8 (93.7; 95.8) | 9.1 (4.2; 16.6) | 98.6 (97.9; 99.1) | 22.1 |
| GOLD 1-4 | 45.5 (28.1; 63.6) | 86.9 (85.3; 88.5) | 6.2 (3.5; 10.0) | 98.8 (98.2; 99.3) | 32.4 |
| FEV_1_/FEV_6_ | 16.7 (5.6; 34.7) | 93.5 (92.2; 94.6) | 4.4 (1.4; 9.9) | 98.4 (97.7; 99.0) | 10.2 |

**Table S3.** Validity parameters for prediction of respiratory mortality for all sites, according to different criteria of Chronic Obstructive Pulmonary Disease (COPD). The PLATINO Study.

| **Diagnostic classification** | **Sensitivity**  **% (95%CI)** | **Specificity**  **% (95%CI)** | **Positive predictive value**  **% (95%CI)** | **Negative predictive value**  **% (95%CI)** | **Youden’s**  **index** |
| --- | --- | --- | --- | --- | --- |
| ***Both Genders*** |  |  |  |  |  |
| LLN | 46.2 (19.2; 74.9) | 87.2 (85.9; 88.4) | 1.6 (0.6; 3.4) | 99.7 (99.4; 99.9) | 33.4 |
| GOLD 2-4 | 26.3 (9.2; 51.2) | 93.5 (92.5; 94.3) | 2.5 (0.8; 5.7) | 99.5 (99.2; 99.7) | 19.8 |
| GOLD 1-4 | 42.1 (20.3; 66.5) | 82.8 (81.4; 84.1) | 1.5 (0.7; 3.0) | 99.6 (99.2; 99.8) | 24.9 |
| FEV_1_/FEV_6_ | 38.5 (13.9; 68.4) | 91.6 (90.5; 92.6) | 2.0 (0.7; 4.6) | 99.7 (99.4; 99.9) | 30.1 |
| ***Males*** |  |  |  |  |  |
| LLN | 55.6 (21.2; 86.3) | 82.4 (80.2; 84.5) | 2.3 (0.8; 5.3) | 99.6 (99.0; 99.9) | 38.0 |
| GOLD 2-4 | 41.7 (15.2; 72.3) | 92.1 (90.4; 93.5) | 4.9 (1.6; 11.1) | 99.4 (98.7; 99.8) | 33.8 |
| GOLD 1-4 | 58.3 (27.7; 84.8) | 77.7 (75.2; 80.0) | 2.5 (1.0; 5.1) | 99.5 (98.8; 99.8) | 36.0 |
| FEV_1_/FEV_6_ | 44.4 (13.7; 78.8) | 89.1 (87.2; 90.8) | 2.9 (0.8; 7.4) | 99.5 (98.9; 99.8) | 33.5 |
| ***Females*** |  |  |  |  |  |
| LLN | 25.0 (0.6; 80.6) | 90.5 (89.0; 91.9) | 0.6 (0.0; 3.4) | 99.8 (99.4; 100.0) | 15.5 |
| GOLD 2-4 | 0.0 (0.0; 41.0) | 94.4 (93.2; 95.4) | 0.0 (0.0; 3.7) | 99.6 (99.1; 99.8) | -5.6 |
| GOLD 1-4 | 14.3 (0.4; 57.9) | 86.3 (84.6; 87.9) | 0.4 (0.0; 2.3) | 99.6 (99.2; 99.9) | 0.6 |
| FEV_1_/FEV_6_ | 25.0 (0.6; 80.6) | 93.4 (92.1; 94.5) | 0.9 (0.0; 4.8) | 99.8 (99.5; 100.0) | 18.4 |

**Table S4.** Validity parameters for prediction of cancer mortality for all sites, according to different criteria of Chronic Obstructive Pulmonary Disease (COPD). The PLATINO Study.

| **Diagnostic classification** | **Sensitivity**  **% (95%CI)** | **Specificity**  **% (95%CI)** | **Positive predictive value**  **% (95%CI)** | **Negative predictive value**  **% (95%CI)** | **Youden’s**  **index** |
| --- | --- | --- | --- | --- | --- |
| ***Both Genders*** |  |  |  |  |  |
| LLN | 11.1 (3.7; 24.1) | 87.0 (85.7; 88.2) | 1.3 (0.4; 3.0) | 98.4 (97.9; 98.9) | -1.9 |
| GOLD 2-4 | 8.7 (2.4; 20.8) | 93.4 (92.4; 94.2) | 2.0 (0.5; 5.0) | 98.5 (98.0; 98.9) | 2.1 |
| GOLD 1-4 | 28.3 (16.0; 43.5) | 82.8 (81.4; 84.1) | 2.5 (1.3; 4.2) | 98.7 (98.1; 99.1) | 11.1 |
| FEV_1_/FEV_6_ | 4.4 (0.5; 15.1) | 91.4 (90.3; 92.4) | 0.8 (0.1; 2.9) | 98.4 (97.9; 98.8) | -4.2 |
| ***Males*** |  |  |  |  |  |
| LLN | 20.0 (4.3; 48.1) | 82.2 (79.9; 84.3) | 1.4 (0.3; 4.0) | 98.8 (97.9; 99.4) | 2.2 |
| GOLD 2-4 | 18.8 (4.1; 45.6) | 91.9 (90.2; 93.4) | 2.9 (0.6; 8.4) | 98.9 (98.1; 99.4) | 10.7 |
| GOLD 1-4 | 37.5 (15.2; 64.6) | 77.5 (75.1; 79.8) | 2.1 (0.8; 4.6) | 99.0 (98.1; 99.5) | 15.0 |
| FEV_1_/FEV_6_ | 13.3 (1.7; 40.5) | 88.8 (86.9; 90.6) | 1.5 (0.2; 5.2) | 98.8 (98.0; 99.4) | 2.1 |
| ***Females*** |  |  |  |  |  |
| LLN | 6.7 (0.8; 22.1) | 90.4 (88.9; 91.8) | 1.2 (0.1; 4.3) | 98.2 (97.4; 98.8) | -2.9 |
| GOLD 2-4 | 3.3 (0.1; 17.2) | 94.4 (93.2; 95.4) | 1.0 (0.0; 5.5) | 98.3 (97.5; 98.8) | -2.3 |
| GOLD 1-4 | 23.3 (9.9; 42.3) | 86.5 (84.8; 88.1) | 2.9 (1.2; 5.8) | 98.5 (97.8; 99.0) | 9.8 |
| FEV_1_/FEV_6_ | 0.0 (0.0; 11.6) | 93.2 (91.9; 94.4) | 0.0 (0.0; 3.2) | 98.1 (97.3; 98.7) | -6.8 |
