# Supplementary material for: FEV1 Is a Better Predictor of Mortality than FVC: The PLATINO Cohort Study
Source: PLoS One. 2014 Oct 6;9(10):e109732. doi: 10.1371/journal.pone.0109732 (PMC4186841; doi:10.1371/journal.pone.0109732)
Supplement: File S3 — Table S5. Best cut-off points of forced expiratory volume in 1 second (FEV1) and forced vital capacity (FVC) to predict overall mortality in individuals belonging to the Platino Study. Table S6. Best cut-off points of forced expiratory volume in 1 second (FEV1) and forced vital capacity (FVC) to predict mortality by cardiovascular diseases in individuals belonging to the Platino Study. Table S7. Best cut-off points of forced expiratory volume in 1 second (FEV1) and forced vital capacity (FVC) to predict mortality by respiratory diseases in individuals belonging to the Platino Study. Table S8. Best cut-off points of forced expiratory volume in 1 second (FEV1) and forced vital capacity (FVC) to predict mortality by cancer in individuals belonging to the Platino Study. (DOC) [file pone.0109732.s003.doc]

**Table S5.** Best cut-off points of forced expiratory volume in 1 second (FEV_1_) and forced vital capacity (FVC) to predict overall mortality in individuals belonging to the Platino Study.

| **Males** | | | | |  | **Females** | | | | |
| --- | --- | --- | --- | --- | --- | --- | --- | --- | --- | --- |
| **Cut-off points** | **Sensitivity (%)** | **Specificity (%)** | **Accuracy (%)** | **Youden’s index** |  | **Cut-off points** | **Sensitivity (%)** | **Specificity (%)** | **Accuracy (%)** | **Youden’s index** |
| **FEV_1_** |  |  |  |  |  | **FEV_1_** |  |  |  |  |
| ≤ 3.2 | 78.9 | 48.2 | 51.0 | 27.1 |  | ≤ 2.4 | 89.9 | 37.4 | 40.7 | 27.3 |
| ≤ 3.1 | 74.3 | 53.7 | 55.5 | 28.0 |  | ≤ 2.3 | 89.0 | 45.7 | 48.4 | 34.7 |
| ≤ 3 | 71.6 | 59.4 | 60.4 | 30.9 |  | ≤ 2.2 | 84.4 | 53.0 | 55.0 | 37.4 |
| ≤ 2.9 | 66.1 | 65.0 | 65.1 | 31.1 |  | **≤ 2.1** | **81.7** | **59.7** | **61.1** | **41.4** |
| **≤ 2.8** | **63.3** | **70.6** | **70.0** | **33.9** |  | ≤ 2 | 71.6 | 66.6 | 66.9 | 38.1 |
| ≤ 2.7 | 57.8 | 75.4 | 73.9 | 33.2 |  | ≤ 1.9 | 64.2 | 72.6 | 72.1 | 36.8 |
| ≤ 2.6 | 54.1 | 79.7 | 77.4 | 33.8 |  | ≤ 1.8 | 60.6 | 77.3 | 76.2 | 37.9 |
| ≤ 2.5 | 48.6 | 84.1 | 80.9 | 32.7 |  | ≤ 1.7 | 55.1 | 82.8 | 81.1 | 37.9 |
| ≤ 2.4 | 45.9 | 87.1 | 83.4 | 33.0 |  | ≤ 1.6 | 45.0 | 86.9 | 84.3 | 31.9 |
| ≤ 2.3 | 39.5 | 88.9 | 84.5 | 28.3 |  | ≤ 1.5 | 40.4 | 90.4 | 87.2 | 30.8 |
| **FVC** |  |  |  |  |  | **FVC** |  |  |  |  |
| ≤ 4 | 58.6 | 57.6 | 57.7 | 16.2 |  | ≤ 2.9 | 84.6 | 46.3 | 48.8 | 30.9 |
| ≤ 3.9 | 53.2 | 63.2 | 62.3 | 16.4 |  | ≤ 2.8 | 80.9 | 52.7 | 54.5 | 33.6 |
| ≤ 3.8 | 46.9 | 67.9 | 66.0 | 14.8 |  | ≤ 2.7 | 73.6 | 59.7 | 60.6 | 33.4 |
| ≤ 3.7 | 43.2 | 71.3 | 68.7 | 14.5 |  | ≤ 2.6 | 70.0 | 65.9 | 66.1 | 35.9 |
| ≤ 3.6 | 42.3 | 75.8 | 72.7 | 18.1 |  | ≤ 2.5 | 65.5 | 71.1 | 70.8 | 36.6 |
| ≤ 3.5 | 38.7 | 79.4 | 75.7 | 18.1 |  | **≤ 2.4** | **61.8** | **75.8** | **74.9** | **37.7** |
| ≤ 3.4 | 35.1 | 83.4 | 79.0 | 18.5 |  | ≤ 2.3 | 52.7 | 80.9 | 79.1 | 33.6 |
| ≤ 3.3 | 31.5 | 87.0 | 81.9 | 18.5 |  | ≤ 2.2 | 46.4 | 85.8 | 83.3 | 32.2 |
| **≤ 3.2** | **29.7** | **89.7** | **84.2** | **19.4** |  | ≤ 2.1 | 40.0 | 89.6 | 86.4 | 29.6 |
| ≤ 3.1 | 27.0 | 91.3 | 85.5 | 18.4 |  | ≤ 2 | 39.1 | 91.5 | 88.2 | 30.6 |

Bold values indicated the Best Cut-off points according Youden’s Index.

**Table S6.** Best cut-off points of forced expiratory volume in 1 second (FEV_1_) and forced vital capacity (FVC) to predict mortality by cardiovascular diseases in individuals belonging to the Platino Study**.**

| **Males** | | | | |  | **Females** | | | | |
| --- | --- | --- | --- | --- | --- | --- | --- | --- | --- | --- |
| **Cut-off points** | **Sensitivity (%)** | **Specificity (%)** | **Accuracy (%)** | **Youden’s index** |  | **Cut-off points** | **Sensitivity (%)** | **Specificity (%)** | **Accuracy (%)** | **Youden’s index** |
| **FEV_1_** |  |  |  |  |  | **FEV_1_** |  |  |  |  |
| ≤ 3 | 77.4 | 57.5 | 58.0 | 34.9 |  | ≤ 2.3 | 93.1 | 44.1 | 44.9 | 37.2 |
| ≤ 2.9 | 74.2 | 63.2 | 63.5 | 37.4 |  | ≤ 2.2 | 93.1 | 51.4 | 52.1 | 44.5 |
| ≤ 2.8 | 74.2 | 68.7 | 68.9 | 42.9 |  | ≤ 2.1 | 93.1 | 58.0 | 58.6 | 51.1 |
| ≤ 2.7 | 71.0 | 73.5 | 73.5 | 44.5 |  | ≤ 2 | 82.8 | 65.0 | 65.3 | 47.7 |
| ≤ 2.6 | 64.5 | 77.7 | 77.4 | 42.2 |  | ≤ 1.9 | 75.9 | 71.0 | 71.1 | 46.9 |
| ≤ 2.5 | 61.3 | 82.3 | 81.7 | 43.5 |  | **≤ 1.8** | **75.9** | **75.8** | **75.8** | **51.6** |
| **≤ 2.4** | **61.3** | **85.3** | **84.7** | **46.6** |  | ≤ 1.7 | 69.0 | 81.3 | 81.1 | 50.2 |
| ≤ 2.3 | 58.1 | 87.5 | 86.7 | 45.6 |  | ≤ 1.6 | 58.6 | 85.6 | 85.2 | 44.3 |
| ≤ 2.2 | 45.2 | 89.9 | 88.8 | 35.1 |  | ≤ 1.5 | 51.7 | 89.1 | 88.5 | 40.9 |
| ≤ 2.1 | 41.9 | 92.3 | 91.0 | 34.3 |  | ≤ 1.4 | 41.4 | 92.1 | 91.3 | 33.5 |
| **FVC** |  |  |  |  |  | **FVC** |  |  |  |  |
| **≤ 4** | **71.9** | **56.9** | **57.3** | **28.8** |  | ≤ 2.8 | 86.7 | 51.2 | 51.8 | 37.9 |
| ≤ 3.9 | 65.6 | 62.5 | 62.6 | 28.1 |  | ≤ 2.7 | 80.0 | 58.3 | 58.6 | 38.3 |
| ≤ 3.8 | 53.1 | 67.2 | 66.8 | 20.3 |  | ≤ 2.6 | 76.7 | 64.3 | 64.5 | 41.0 |
| ≤ 3.7 | 46.9 | 70.4 | 69.8 | 17.3 |  | ≤ 2.5 | 73.3 | 69.5 | 69.6 | 42.9 |
| ≤ 3.6 | 46.9 | 74.7 | 73.9 | 21.5 |  | **≤ 2.4** | **73.3** | **74.3** | **74.3** | **47.6** |
| ≤ 3.5 | 37.5 | 78.1 | 77.1 | 15.6 |  | ≤ 2.3 | 60.0 | 79.4 | 79.1 | 39.4 |
| ≤ 3.4 | 34.4 | 82.1 | 80.8 | 16.5 |  | ≤ 2.2 | 53.3 | 84.4 | 83.9 | 37.8 |
| ≤ 3.3 | 34.4 | 85.8 | 84.5 | 20.2 |  | ≤ 2.1 | 50.0 | 88.4 | 87.7 | 38.4 |
| ≤ 3.2 | 34.4 | 88.5 | 87.1 | 22.9 |  | ≤ 2 | 50.0 | 90.3 | 89.6 | 40.3 |
| ≤ 3.1 | 34.4 | 90.3 | 88.8 | 24.7 |  | ≤ 1.9 | 43.3 | 92.9 | 92.1 | 36.3 |

Bold values indicated the Best Cut-off points according Youden’s Index.

**Table S7.** Best cut-off points of forced expiratory volume in 1 second (FEV_1_) and forced vital capacity (FVC) to predict mortality by respiratory diseases in individuals belonging to the Platino Study.

| **Males** | | | | |  | **Females** | | | | |
| --- | --- | --- | --- | --- | --- | --- | --- | --- | --- | --- |
| **Cut-off points** | **Sensitivity (%)** | **Specificity (%)** | **Accuracy (%)** | **Youden’s index** |  | **Cut-off points** | **Sensitivity (%)** | **Specificity (%)** | **Accuracy (%)** | **Youden’s index** |
| **FEV_1_** |  |  |  |  |  | **FEV_1_** |  |  |  |  |
| ≤ 2.8 | 77.8 | 68.0 | 68.0 | 45.8 |  | ≤ 2.4 | 100.0 | 35.8 | 35.9 | 35.8 |
| ≤ 2.7 | 66.7 | 72.7 | 72.7 | 39.4 |  | ≤ 2.3 | 100.0 | 43.6 | 43.7 | 43.6 |
| ≤ 2.6 | 66.7 | 76.9 | 76.9 | 43.6 |  | ≤ 2.2 | 100.0 | 50.7 | 50.8 | 50.7 |
| ≤ 2.5 | 66.7 | 81.5 | 81.4 | 48.2 |  | ≤ 2.1 | 100.0 | 57.3 | 57.4 | 57.3 |
| ≤ 2.4 | 55.6 | 84.4 | 84.2 | 40.0 |  | ≤ 2 | 100.0 | 64.3 | 64.4 | 64.3 |
| ≤ 2.3 | 55.6 | 86.6 | 86.4 | 42.2 |  | ≤ 1.9 | 100.0 | 70.4 | 70.5 | 70.4 |
| ≤ 2.2 | 55.6 | 89.4 | 89.1 | 44.9 |  | ≤ 1.8 | 100.0 | 75.1 | 75.1 | 75.1 |
| ≤ 2.1 | 55.6 | 91.8 | 91.5 | 47.3 |  | **≤ 1.7** | **100.0** | **80.6** | **80.7** | **80.6** |
| ≤ 2 | 55.6 | 93.7 | 93.4 | 49.3 |  | ≤ 1.6 | 75.0 | 85.0 | 85.0 | 60.0 |
| **≤ 1.9** | **55.6** | **94.8** | **94.5** | **50.3** |  | ≤ 1.5 | 50.0 | 88.5 | 88.4 | 38.5 |
| **FVC** |  |  |  |  |  | **FVC** |  |  |  |  |
| ≤ 3.9 | 66.7 | 62.0 | 62.0 | 28.6 |  | ≤ 2.9 | 100.0 | 44.5 | 44.6 | 44.5 |
| ≤ 3.8 | 66.7 | 66.9 | 66.9 | 33.5 |  | ≤ 2.8 | 100.0 | 50.6 | 50.8 | 50.6 |
| ≤ 3.7 | 66.7 | 70.3 | 70.2 | 36.9 |  | ≤ 2.7 | 100.0 | 57.7 | 57.8 | 57.7 |
| ≤ 3.6 | 66.7 | 74.4 | 74.3 | 41.1 |  | ≤ 2.6 | 100.0 | 63.7 | 63.8 | 63.7 |
| ≤ 3.5 | 66.7 | 78.0 | 78.0 | 44.7 |  | ≤ 2.5 | 100.0 | 69.0 | 69.0 | 69.0 |
| ≤ 3.4 | 66.7 | 82.0 | 81.9 | 48.7 |  | ≤ 2.4 | 100.0 | 73.6 | 73.7 | 73.6 |
| **≤ 3.3** | **66.7** | **85.7** | **85.5** | **52.3** |  | ≤ 2.3 | 100.0 | 78.9 | 78.9 | 78.9 |
| ≤ 3.2 | 55.6 | 88.2 | 88.0 | 43.8 |  | **≤ 2.2** | **100.0** | **84.0** | **84.0** | **84.0** |
| ≤ 3.1 | 55.6 | 90.0 | 89.7 | 45.5 |  | ≤ 2.1 | 75.0 | 87.9 | 87.8 | 62.9 |
| ≤ 3 | 55.6 | 91.6 | 91.3 | 47.1 |  | ≤ 2 | 50.0 | 89.7 | 89.6 | 39.7 |

Bold values indicated the Best Cut-off points according Youden’s Index.

**Table S8.** Best cut-off points of forced expiratory volume in 1 second (FEV_1_) and forced vital capacity (FVC) to predict mortality by cancer in individuals belonging to the Platino Study.

| **Males** | | | | |  | **Females** | | | | |
| --- | --- | --- | --- | --- | --- | --- | --- | --- | --- | --- |
| **Cut-off points** | **Sensitivity (%)** | **Specificity (%)** | **Accuracy (%)** | **Youden’s index** |  | **Cut-off points** | **Sensitivity (%)** | **Specificity (%)** | **Accuracy (%)** | **Youden’s index** |
| **FEV_1_** |  |  |  |  |  | **FEV_1_** |  |  |  |  |
| ≤ 3 | 53.3 | 56.7 | 56.7 | 10.0 |  | ≤ 2.4 | 80.0 | 36.0 | 36.7 | 16.0 |
| ≤ 2.9 | 53.3 | 62.5 | 62.4 | 15.8 |  | **≤ 2.3** | **80.0** | **43.9** | **44.5** | **23.9** |
| ≤ 2.8 | 46.7 | 67.8 | 67.6 | 14.5 |  | ≤ 2.2 | 70.0 | 51.0 | 51.3 | 21.0 |
| ≤ 2.7 | 46.7 | 72.6 | 72.3 | 19.3 |  | ≤ 2.1 | 63.3 | 57.5 | 57.6 | 20.8 |
| **≤ 2.6** | **46.7** | **76.9** | **76.5** | **23.6** |  | ≤ 2 | 53.3 | 64.5 | 64.3 | 17.8 |
| ≤ 2.5 | 26.7 | 81.2 | 80.6 | 7.9 |  | ≤ 1.9 | 40.0 | 70.4 | 69.9 | 10.4 |
| ≤ 2.4 | 26.7 | 84.2 | 83.5 | 10.9 |  | ≤ 1.8 | 40.0 | 75.2 | 74.6 | 15.2 |
| ≤ 2.3 | 26.7 | 86.5 | 85.8 | 13.2 |  | ≤ 1.7 | 40.0 | 80.8 | 80.1 | 20.8 |
| ≤ 2.2 | 20.0 | 89.2 | 88.3 | 9.2 |  | ≤ 1.6 | 30.0 | 85.2 | 84.2 | 15.2 |
| ≤ 2.1 | 20.0 | 91.6 | 90.7 | 11.6 |  | ≤ 1.5 | 26.7 | 88.7 | 87.6 | 15.4 |
| **FVC** |  |  |  |  |  | **FVC** |  |  |  |  |
| ≤ 5.3 | 100.0 | 8.2 | 9.3 | 8.2 |  | ≤ 2.9 | 76.7 | 44.8 | 45.3 | 21.4 |
| ≤ 5.2 | 100.0 | 10.2 | 11.3 | 10.2 |  | ≤ 2.8 | 73.3 | 50.9 | 51.3 | 24.3 |
| ≤ 5.1 | 100.0 | 13.1 | 14.1 | 13.1 |  | **≤ 2.7** | **66.7** | **58.0** | **58.2** | **24.7** |
| **≤ 5** | **100.0** | **16.1** | **17.1** | **16.1** |  | ≤ 2.6 | 56.7 | 63.9 | 63.8 | 20.6 |
| ≤ 4.9 | 93.3 | 18.4 | 19.3 | 11.7 |  | ≤ 2.5 | 50.0 | 69.1 | 68.8 | 19.1 |
| ≤ 4.8 | 80.0 | 21.6 | 22.3 | 1.6 |  | ≤ 2.4 | 43.3 | 73.7 | 73.2 | 17.1 |
| ≤ 4.7 | 73.3 | 24.9 | 25.5 | -1.8 |  | ≤ 2.3 | 36.7 | 79.0 | 78.3 | 15.7 |
| ≤ 4.6 | 73.3 | 29.3 | 29.9 | 2.6 |  | ≤ 2.2 | 30.0 | 84.0 | 83.1 | 14.0 |
| ≤ 4.5 | 66.7 | 32.6 | 33.1 | -0.7 |  | ≤ 2.1 | 26.7 | 88.0 | 86.9 | 14.6 |
| ≤ 4.4 | 66.7 | 37.5 | 37.8 | 4.1 |  | ≤ 2 | 26.7 | 89.9 | 88.8 | 16.5 |

Bold values indicated the Best Cut-off points according Youden’s Index.
