# Supplementary material for: FEV1 Is a Better Predictor of Mortality than FVC: The PLATINO Cohort Study
Source: PLoS One. 2014 Oct 6;9(10):e109732. doi: 10.1371/journal.pone.0109732 (PMC4186841; doi:10.1371/journal.pone.0109732)
Supplement: File S4 — Table S9. Hazard ratio between Chronic Obstructive Pulmonary Disease criteria and mortality in all sites from the PLATINO Study, except Montevideo. (DOC) [file pone.0109732.s004.doc]

**Table S9.** Hazard ratio between Chronic Obstructive Pulmonary Disease criteria and mortality in all sites from the PLATINO Study, except Montevideo.

|  | **Cardiovascular** | | **Respiratory** | | **Neoplasia** | |
| --- | --- | --- | --- | --- | --- | --- |
|  | **Crude*** | **Adjusted**** | **Crude*** | **Adjusted**** | **Crude*** | **Adjusted**** |
|  | **HR (95% CI)** | **HR (95% CI)** | **HR (95% CI)** | **HR (95% CI)** | **HR (95% CI)** | **HR (95% CI)** |
| ***Both Genders*** |  |  |  |  |  |  |
| LLN | 1.85 (1.00; 3.42) | 1.45 (0.74; 2.84) | 4.79 (1.36; 16.90) | 3.24 (0.96; 10.93) | 0.79 (0.30; 2.06) | 0.65 (0.24; 1.77) |
| GOLD II-IV | 2.78 (1.39; 5.55) | 2.35 (1.17; 4.73) | 2.56 (0.85; 7.75) | 0.98 (0.30; 3.19) | 1.14 (0.40; 3.24) | 0.99 (0.36; 2.76) |
| GOLD 1-4 | 2.18 (1.31; 3.62) | 1.74 (1.02; 2.98) | 1.82 (0.77; 4.31) | 1.00 (0.39; 2.56) | 1.56 (0.79; 3.06) | 1.40 (0.70; 2.78) |
| FEV1/FEV6<LLN | 2.23 (1.11; 4.47) | 1.78 (0.90; 3.53) | 4.35 (1.16; 16.32) | 3.31 (0.86; 12.77) | 0.47 (0.11; 2.05) | 0.40 (0.10; 1.67) |
| ***Males*** |  |  |  |  |  |  |
| LLN | 2.40 (1.14; 5.05) | 2.05 (0.92; 4.57) | 4.47 (1.29; 15.54) | 2.42 (0.84; 6.98) | 0.57 (0.12; 2.65) | 0.51 (0.10; 2.47) |
| GOLD II-IV | 3.43 (1.38; 8.51) | 3.71 (1.38; 9.99) | 5.64 (1.55; 20.49) | 2.38 (0.69; 8.21) | 1.21 (0.27; 5.34) | 1.04 (0.25; 4.33) |
| GOLD 1-4 | 2.27 (1.02; 5.05) | 1.75 (0.73; 4.21) | 3.52 (1.06; 11.73) | 1.79 (0.44; 7.32) | 1.38 (0.48; 4.00) | 1.39 (0.51; 3.83) |
| FEV1/FEV6<LLN | 3.34 (1.46; 7.60) | 2.99 (1.35; 6.63) | 4.11 (1.00; 16.88) | 2.42 (0.77; 7.67) | 0.46 (0.05; 3.95) | 0.41 (0.05; 3.15) |
| ***Females*** |  |  |  |  |  |  |
| LLN | 1.13 (0.40; 3.17) | 0.77 (0.24; 2.55) | 1.38 (0.23; 8.26) | 4.74 (0.74; 30.53) | 1.05 (0.30; 3.60) | 0.95 (0.26; 3.40) |
| GOLD II-IV | 2.15 (0.80; 5.80) | 1.71 (0.65; 4.52) |  |  | 1.06 (0.24; 4.75) | 1.00 (0.25; 3.99) |
| GOLD 1-4 | 1.99 (1.02; 3.91) | 1.65 (0.82; 3.33) | 0.38 (0.12; 1.18) | 0.14 (0.02; 0.75) | 1.72 (0.70; 4.23) | 1.62 (0.66; 4.01) |
| FEV1/FEV6<LLN | 1.07 (0.31; 3.68) | 0.62 (0.15; 2.53) | 1.45 (0.24; 8.56) | 4.47 (1.03; 19.38) | 0.50 (0.06; 4.05) | 0.44 (0.06; 3.24) |

* Adjusted for age and country

** Adjusted for age, country, schooling, smoking status, Pack-years, quality of life, BMI and comorbidities score.
